# Duodenal lymphangioma with extensive edema of gastric antrum mucosa

**DOI:** 10.1055/a-2432-3454

**Published:** 2024-10-25

**Authors:** Wenlei Li, Junbin Yan, Jianyu Lv, Yuxuan Chen, Siyi Ni, Shuo Zhang

**Affiliations:** 1 The First Clinical College, Zhejiang Chinese Medical University, Hangzhou, China; 2 70571 The Second Clinical College, Zhejiang Chinese Medical University, Hangzhou, China; 3 587400 Department of Gastroenterology, The Second Affiliated Hospital of Zhejiang Chinese Medical University, Hangzhou, China

A 37-year-old woman was referred to our institution following the discovery of a gastric submucosal tumor. Abdominal contrast-enhanced computed tomography revealed diffuse thickening of the gastric antrum wall and a hypodense perigastric lesion ( Fig. 1 ). Abdominal enhanced magnetic resonance imaging demonstrated submucosal lesions in the gastric antrum and descending duodenal bulb, along with a cystic lesion between the perigastric and lesser omentum ( Fig. 2 ). Esophagogastroduodenoscopy indicated mucosal uplift in the gastric antrum ( Fig. 3 **a** ), and a large oval uplift on the side of the gastric small curvature, measuring approximately 5.0 × 4.0 cm ( Fig. 3 **b** ). Clusters of white granules were observed on the mucosa of the duodenal bulb ( Fig. 3 **c** ). Endoscopic ultrasonography revealed hypoechoic lesions with visible space, thickening of the cystic wall locally, with the largest interface measuring about 5.7 × 4.6 cm ( Fig. 4 ). Fluid was aspirated from the diseased gastric antrum mucosa for examination, and a biopsy of the duodenal lesions was performed ( Fig. 3 **d** , Video 1 ). Pathological examination confirmed lymphangioma ( Fig. 5 ).

**Fig. 1:**
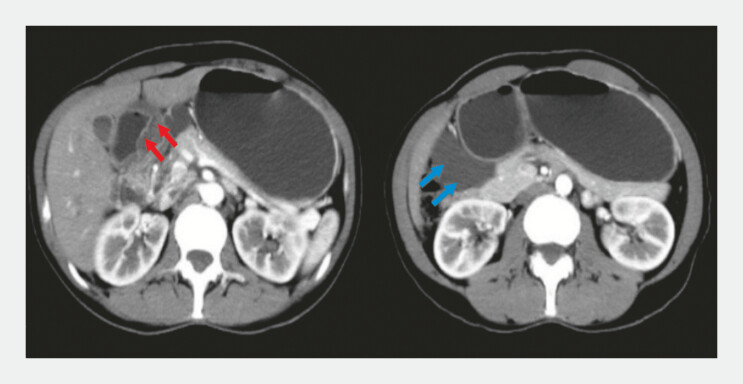
Full abdominal computed tomography with contrast enhancement. Diffuse thickening of the gastric antrum wall (red arrow). Hypodense perigastric focus (blue arrow).

**Fig. 2:**
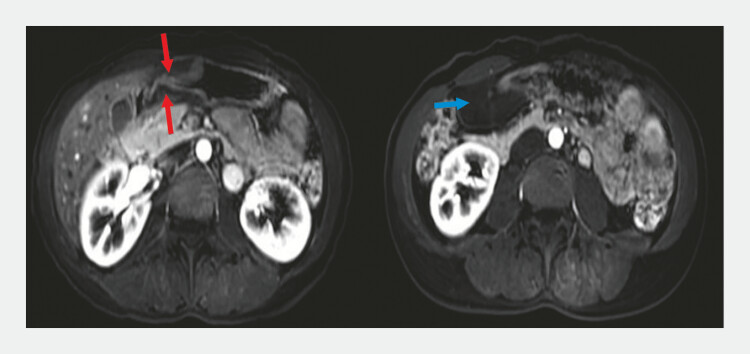
Abdominal enhanced magnetic resonance imaging scan. Submucosal lesions in the gastric antrum and descending duodenal bulb (red arrow). Cystic lesion between the perigastric and lesser omentum (blue arrow).

**Fig. 3:**
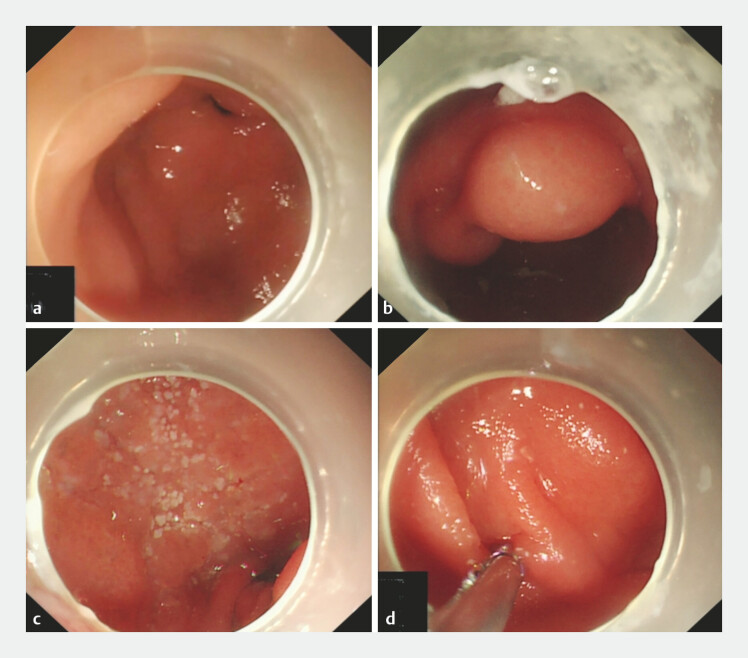
Gastroscopy images. **a** Uplift of the gastric antrum mucosa. **b** Large oval uplift on the side of the gastric small curvature, measuring approximately 5.0 × 4.0 cm. **c** Clustered white granules on the mucosa of the duodenal bulb. **d** Biopsy of the duodenal lesions.

**Fig. 4:**
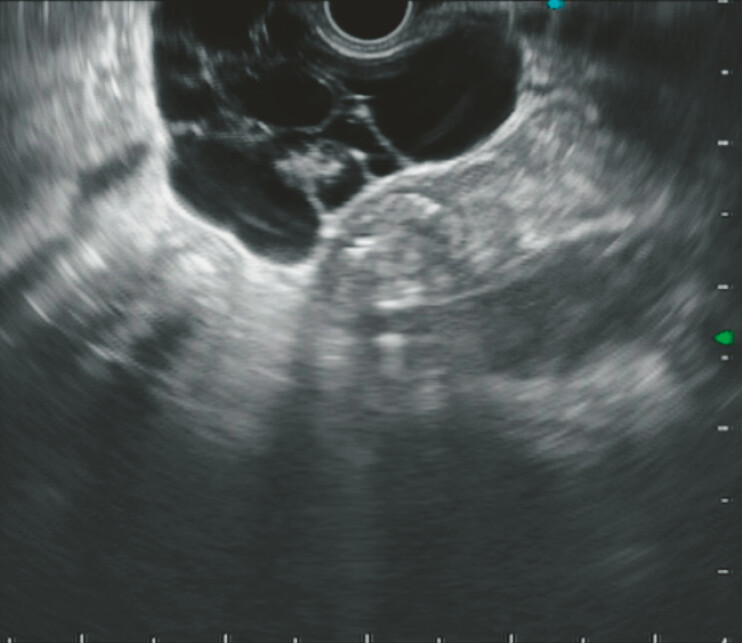
Endoscopic ultrasonography. Hypoechoic lesions, visible space through the stomach lining, and local cystic wall thickening; largest interface measured approximately 5.7 × 4.6 cm.

**Fig. 5:**
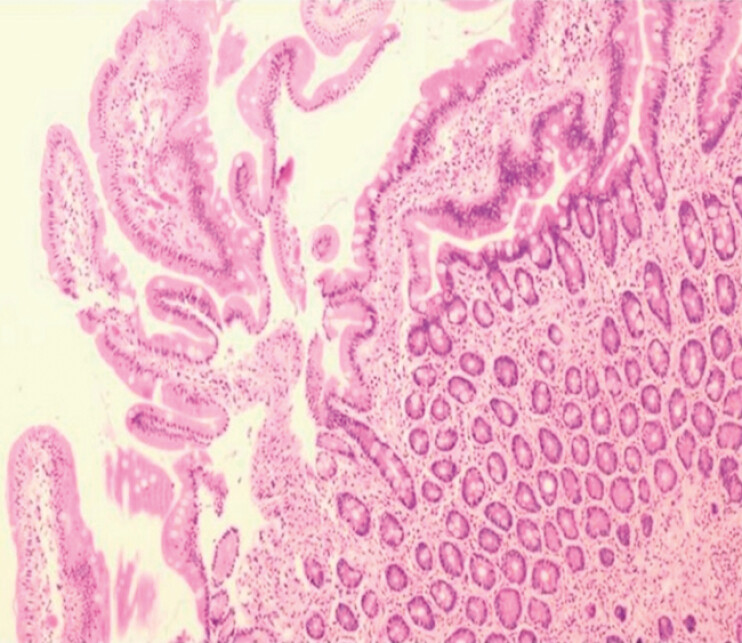
Pathological image (hematoxylin and eosin staining,10 × 4 magnification). Duodenal mucosa showing chronic inflammation with lymphangiectasia.

Large oval uplift on the side of the gastric small curvature.Video 1

Significantly elevated levels of tumor markers (carcinoembryonic antigen, carbohydrate antigen 19–9, carbohydrate antigen 125, cytokeratin fragment 21–1) were noted in the lymphatic fluid, while corresponding oncological markers in the serum showed no significant abnormalities ( Table 1 ).

**Table:** **Table 1** Tumor marker levels in puncture fluid and blood.

Marker	Puncture fluid	Blood	Reference range (blood)
AFP	0.7	1.6	0–8 ng/mL
CEA	211.3 ↑	1.2	0–5 ng/mL
CA19–9	418.7 ↑	26.1	0–37 U/mL
CA125	181.8 ↑	23.2	0–35 U/mL
CA15–3	6.6	10.5	0–31.3 U/mL
CYFRA21–1	9.35↑	0.99	0–2.08 ng/mL
SCCAg	0.4	0.4	<1.5 ug/L
AFP, alpha fetoprotein; CA, carbohydrate antigen; CEA, carcinoembryonic antigen; CYFRA21–1, cytokeratin fragment 21–1; SCCAg, squamous cell carcinoma. antigen.			

Duodenal lymphangioma is characterized by the dilation of duodenal structures and proliferation of endothelial cells within lymphatic vessels and connective tissue, typically presenting as benign. Therefore, intervention is usually warranted only in the presence of clinical manifestations such as infection, fistulization, or hemorrhage 1 2 . We report a rare case of massive duodenal lymphangioma, accompanied by prominent gastric antrum mucosal edema, for academic discussion. Despite normal serum oncological markers, a significant elevation of tumor markers in lymphatic fluid suggests a potential risk of malignant transformation. Thus, our understanding of duodenal lymphangioma warrants further enhancement. Patients with massive duodenal lymphangiomas require regular follow-up.

Endoscopy_UCTN_Code_CCL_1AB_2AC_3AB

## Notes

Endoscopy E-Videos https://eref.thieme.de/e-videos
*E-Videos* is an open access online section of the journal *Endoscopy* , reporting on interesting cases and new techniques in gastroenterological endoscopy. All papers include a high-quality video and are published with a Creative Commons CC-BY license. Endoscopy E-Videos qualify for HINARI discounts and waivers and eligibility is automatically checked during the submission process. We grant 100% waivers to articles whose corresponding authors are based in Group A countries and 50% waivers to those who are based in Group B countries as classified by Research4Life (see: https://www.research4life.org/access/eligibility/ ). This section has its own submission website at https://mc.manuscriptcentral.com/e-videos .
